# Combination of androgen and estrogen improves asthma by mediating Runx3 expression

**DOI:** 10.7150/ijms.91253

**Published:** 2024-04-08

**Authors:** Yi He, Binaya Wasti, Yu Yuan, Zhifeng Chen, Wentao Duan, Jingsi Jia, Bing Xiao, Xiufeng Zhang, Jianmin Li, Qingping Zeng, Libing Ma, Shaokun Liu, Xudong Xiang

**Affiliations:** 1 Department of Respiratory and Critical Care Medicine, The Second Xiangya Hospital, Central South University, 139 Middle Renmin Road, Changsha, Hunan 410011, China.; 2 Department of Emergency Medicine, The Second Xiangya Hospital, Central South University, Emergency and Difficult Diseases Institute of Central South University, 139 Middle Renmin Road, Changsha, Hunan 410011, China.; 3 Department of Respiratory Medicine, The Second Affiliated Hospital of Hainan Medical University, 48 Pak Shui Tong Road, Haikou, Hainan 570000, China.; 4 Department of Respiratory and Critical Care Medicine, Hunan Provincial People's Hospital, 61 West Jiefang Road, Changsha, Hunan 410005, China.; 5 Department of Respiratory and Critical Care Medicine, Longshan County People's Hospital, Longshan, Hunan 416800, China.; 6 Department of Respiratory and Critical Care Medicine, The Affiliated Hospital of Guilin Medical University, 15 Le Qun Road, Guilin, Guangxi 541001, China.

**Keywords:** androgen, estrogen, asthma, Runx3

## Abstract

**Objective:** Asthma is a chronic heterogeneous airway disease, and imbalanced T-helper type 1 (Th1) and Th2 cell-mediated inflammation contribute to its pathogenesis. Although it has been suggested that androgen and estrogen were involved in development of asthma, the underlying mechanisms remained largely unclear. Studies have demonstrated that Runx3 could promote naive CD4^+^ T cells to differentiate into Th1 cells. Hence, our study aimed to explore the potential regulatory mechanism of androgen and estrogen on asthma via modulating Runx3.

**Methods:** First, clinical assessments and pulmonary function tests were conducted on 35 asthma patients and 24 healthy controls. The concentrations of androgen, estrogen, and androgen estrogen ratios were assessed in peripheral blood samples of asthma patients and healthy controls. Then, a murine asthma model was established to explore the effects of estrogen and androgen (alone or in combination) on asthma. Third, an in vitro assay was used to explore the mechanism of combination of androgen and estrogen in asthma.

**Results:** We observed decreased androgen and increased estrogen levels in asthma patients compared with healthy controls. In mice with experimental asthma, there were increased serum concentrations of estrogen and decreased serum concentrations of androgen, intervention with combination of androgen and estrogen alleviated airway inflammations, increased Runx3 expressions and elevated Th1 differentiation. In CD4+ T cells co-cultured with bronchial epithelial cells (BECs), treatment with androgen plus estrogen combination promoted Th1 differentiation, which was mitigated by Runx3 knockdown in BECs and enhanced by Runx3 overexpression.

**Conclusion:** These findings suggest that androgen estrogen combination modulate the Th1/Th2 balance via regulating the expression of Runx3 in BECs, thereby providing experimental evidence supporting androgen and estrogen combination as a novel therapy for asthma.

## 1. Introduction

Asthma is a chronic heterogeneous airway disease with multiple endotypes and phenotypes, characterized by airway inflammation, airway hyperresponsiveness (AHR), and reversible or variable respiratory airflow limitation 1. The prevalence of asthma has risen drastically during the last two decades, and there are significant gender differences in different age groups 2. This gender difference of asthma prevalence may be related to sex hormones (estrogens, progesterone, and androgens) 3. During the menstrual cycle, estrogen and progesterone fluctuation could decrease forced expiratory volume in the first second (FEV1) and forced vital capacity (FVC), increase AHR and asthma severity 4. Moreover, asthma symptoms during pregnancy, the perimenopausal and menopause periods are prone to exacerbate 5. Estrogen (E2) could promote airway inflammation 6, and progesterone could worsen AHR 7, while androgens such as dihydrotestosterone (DHT) could reduce airway inflammation and promote airway smooth muscle relaxation 8-10. However, the underlying mechanisms for the roles of sex hormones in asthma remain largely unclear.

Studies have shown that an imbalance in the T-helper-type 1 (Th1) cells /Th2 cells immune responses is an important factor in the pathogenesis of asthma 11. Type 2(T2) inflammation plays a dominant role in T2-high asthma phenotype, characterized by increased Th2-dependent cytokine such as interleukin-4 (IL-4), interleukin-5 (IL-5), and interleukin-13 (IL-13) 12. On the other hand, Th1 cells could secret IFN-γ and TNF-α, which restrain the maturation of Th2 cells. Therefore, Th1 cells could either contribute to resolution of airway inflammation, or the emergence of “Type-2-low” phenotype of asthma, which is usually more resistant to corticosteroid treatment and not sensitive to monoclonal antibody therapies 11. The naive CD4^+^T cells differentiate into Th1 cells or Th2 cells with the immune stimuli provided by antigen-presenting cells (APCs). Our recent researches have suggested that bronchial epithelial cells (BECs) could act as non-canonical APCs and modulate the differentiation of naïve T cells 13-15, and it had been reported that sex hormones could modulate innate and adaptive immune responses through regulation of dendritic cells (DCs) and resident macrophages 4, 16, yet it is unknown whether sex hormones could influence airway inflammation via BECs.

Runx3 (Runt-related transcription factor 3) is a member of the Runs family proteins which contain a runt domain in the sequences and play essential roles in T cell functions 17. Runx3 participates in the development of immune system and is involved in the differentiation of T cells 18. Runx3 not only promotes the differentiation of naïve CD4+ T cells into Th1 cells but also inhibits the production of IL-4, the kernel cytokine secreted by Th2 cells, thereby regulating the balance of Th1/Th2 cells and the airway inflammatory responses in asthma 19, 20.

In the present study we explored the role and mechanisms of sex hormones in the pathogenesis of asthma. To sum up our results, we have found that combination of androgen and estrogen could alleviate asthmatic airway inflammation through modulation of Runx3 expression in bronchial epithelial cells.

## 2. Materials and Methods

### 2.1. Clinical samples

Asthma patients (n = 35) and healthy controls (HCs, n = 24) were enrolled at the Second Xiangyi Hospital of Central South University. Patients with asthma were diagnosed by a physician according to Global Initiative for Asthma guidelines (GINA). All asthma patients were included with following criteria:1) symptomatic asthma (including wheezing, dyspnea, chest tightness, or coughing); 2) bronchodilator responsiveness (FEV1 change > 200ml and FEV1%predicted improvement > 12%); and /or 3) FEV1%predicted < 60%; 4) had not received inhaled or oral corticosteroid or leukotriene antagonist treatment in the last 3 months; and 5) age≧14 years old. The exclusion criteria were the following: 1) malignant tumors; 2) autoimmune diseases; 3) pregnant and lactating women; and 4) age < 14 years old. Asthma patients were enrolled from visitors of the outpatient department and healthy control participants were enrolled from visitors of the health management center of the hospital. Demographic information (Table 1) and blood samples of the 59 voluntarily enrolled participants were collected for study. The study was approved by the Ethical Review Committee of Second Xiangyi Hospital of Central South University (protocol code LYF2021172 and date of approval 2021/12/15). All enrolled participants signed informed consent agreements.

### 2.2. Mice

Female C57BL/6 mice (aged 6-8 weeks, weighing 18-20g) were housed in the hospital animal center in a controlled environment (temperature 21±2°C, humidity 60±10%, with a 12/12 h light/dark cycle) with free access to standard rodent chow and water. All studies were performed in compliance with the Second Xiangyi Hospital and Central South University Animal Care and Use Committee guidelines (protocol code 2021967 and date of approval 2021/12/29). All mice were allowed to acclimate for three days before treatments. Each mouse was assigned an identification number and randomized to different groups so that all experiments were carried out in a blinded manner. Experiments were conducted during the light phase.

### 2.3. Murine Asthma Model and grouping

A murine asthma model was established in accordance with our previous reports 14, 15. In brief, mice mere intraperitoneally (i.e.) injected with ovalbumin (OVA, Sigma Aldrich, 1mg/mL, 100 ug per animal) and aluminum hydroxide (Sigma Aldrich, 10mg/mL, 2mg per animal) at day-0, day-7, and day-14, followed with daily inhalation of nebulized ovalbumin solution (6%) for 30 minutes from day-10 to day-20. Mice in the blank group received intraperitoneal normal saline injection and nebulized normal saline inhalation. DHT and E2 (Apex BIO) were first dissolved with 2mL of DMSO (Sigma Aldrich) first and then diluted with corn oil to a final concentration of 2mg/mL in accordance with the manufacturer's protocol. The dosage of DHT and/or E2 (30mg/kg) was adopted in accordance to previous reports with minor modification 14, 21. On day 21, mice were anesthetized with intraperitoneal injection of 10% chloral hydrate and northen euthanized with cervical dislocation maneuver. Mice were randomly divided into six groups(n=6/group): blank group [no DHT, no E2, no oil, no saline], asthma group (i.e. injection of saline, no DHT, no E2, no corn oil), asthma normal control (nc) group (i.e. injection of corn oil plus DMSO, no DHT, no E2, no saline), asthma+DHT group (i.e. injection of DHT, no E2, no saline), asthma+E2 group (i.e. injection of E2, no DHT, no saline) and asthma+DHT+E2 group (i.e. injection of DHT and E2, no saline).

### 2.4. Analyses of bronchoalveolar lavage fluid (BALF)

BALF was collected according to a method described previously 22. The trachea was intubated with a venous catheter and the lungs were washed with 0.5 mL saline, and BALF was obtained by three continuous aspirations. The BALF was centrifuged at 1500 rpm for 5 min at 4°C and cell pellets were resuspended in cold PBS. Then cells were fixed and stained using Wright-Giemsa protocol. Differential cell count was performed under a light microscope, 200 cells were count in each slide.

### 2.5. Histopathology

The left lobes of mouse lungs were perfused with 10% formalin via trachea and then isolated and stored in 10% formalin. Paraffin-embedded 5-μm lung sections were stained with hematoxylin and eosin (H&E) and immunohistochemistry (Runx3 antibody (Abcam, Cambridge, USA), ECP antibody (Biorobot, Cambridge, United Kingdom)). Stained sections were selected from each group, and the expression of Runx3 and ECP proteins was assessed by two pathologists blinded to the treatment using a computer image processing system (Image-Pro Plus 6.0). The H&E sections were analyzed for inflammation with a semi-quantification scoring system based on cell count in the peri-bronchial regions in accordance to previous study 17. The scoring system was: 0, no cells; 1, a few cells; 2, single layer of inflammatory cells infiltrated around the bronchus; 3, 2-4 layers of inflammatory cells infiltrated around the bronchus; 4, more than 4 layers of inflammatory cells infiltrated around the bronchus. For IHC staining, five regions of interest (ROIs) were selected in each section, and quantification of Runx3 and ECP expressions were achieved with the integrated optical density (IOD) parameter, which was automatically calculated by the image processing software, the mean IOD of the five ROIs was used to represent the expression level of the corresponding protein in each section.

### 2.6. Bronchial epithelial cells isolation and culture

Bronchial epithelial cells (BECs) were isolated from untreated healthy mice using an improved version of the protocol previously reported 14, 23. Briefly, bronchi were removed from gross anatomy of mice, and the tracheae were dissected lengthways, washed with PBS, and transferred to minimal essential medium (MEM, 11095-080, Fisher Scientific International) preheated to 37 °C containing 0.1 mg/ml DNase and 1.4 mg/ml promise (Roche Diagnostics). After incubation at 37 °C for 1h, the tube containing the tracheae was carefully inverted 12 times to separate the epithelial cells from the airways. 1ml of sterile fetal bovine serum (FBS, Gibco, Australia) was added to stop enzyme digestion. After that, a 150-mesh cell sieve (Bio sharp, BS-100-XBS, China) was used to remove undigested excess tissue. After centrifugation for 5 min at 800×g, the supernatant was discarded and the cells were resuspended with MEM containing 10% FBS. The cells were then inoculated in culture bottles and left standing at 37°C with 5% CO_2_ for 2 h to remove contaminated non-epithelial cells. Subsequently, the culture medium (including suspended cells) was collected and the supernatant was removed after centrifugation at 800×g for 5 min. BECs were cultured with bronchial epithelial growth medium (Procell, CM-M007, China) in a humidified incubator at 37°C with 5% CO_2_. BECs were identified with immunofluorescence staining for pan-cytokeratin (PCK), an epithelial marker. Purity greater than 90% was considered acceptable according to previous report 24.

### 2.7. BECs transfection and grouping

The BECs were transfected with small interfering RNA targeting Runx3 (si-RUNX3) or negative control (is-NC, RI bobo, China) or Overexpression Runx3 (OE-RUNX3) plasmids or negative control (OE-NC, Honor Gene, China) using Lipofectamine 3000 (P3000, Invitrogen) for 48h according to the manufacturer's protocol, and then were treated with 100ug OVA for 24h and finally harvested for protein extraction. In Runx3-Silencing experiments, we set 7 groups, including blank group, asthma group, asthma+DHT/E2 group, asthma+si-NC group, asthma+si-RUNX3 group, asthma+si-NC+DHT/E2 group, asthma+si-RUNX3+DHT/E2 group. In Runx3-Overexpression experiments, we set 8 groups, including blank group, asthma group, asthma+DHT/E2 group, asthma+P3000 group, asthma+OE-NC group, asthma+OE-RUNX3 group, asthma+OE-NC +DHT/E2 group, asthma+OE-RUNX3+DHT/E2 group.

### 2.8. BEC and CD4+ T cell co-culture assay

Magnetic bead separation (130-049-201, Miltenyi Biotec, Germany) were utilized to isolate mice spleen CD4^+^ T cells from untreated healthy mice using the protocol reported in our previous studies 14, 25. BECs were pretreated with 1 nM of E2, 20 nM of DHT and 20:1 nM of DHT:E2 in the asthma+E2, asthma+DHT, asthma+DHT+E2 groups for 24h, respectively. Then BECs were cocultured with CD4+ T cells at a ratio of 10: 1 (TCs: BECs) 13 for 24h with complete RPMI 1640 culture medium (Gibco, Australia) supplemented with 10% FBS, 1% penicillin and streptomycin, soluble anti-CD28 (1.0 μg/ml, eBioscience), soluble anti-CD3 (0.5 μg/ml, eBioscience), and IL-2 (20 ng/ml, eBioscience) in transwell plate (Corning, USA). After 24h, cells were collected and then detected by flow cytometry.

### 2.9. Flow cytometry of CD4+ T cells

CD4+ T cells were stimulated with 2µl/ml (about 1 × 10^6^ cells /ml) of leukocyte activation cocktail (BD Biosciences) and collected after cultured at 37°C and 5% CO2 for 4-6h. First, cells were stained at room temperature for 15 minutes in the dark with a cell viability marker (Fixable Viability Stain 510 antibody, BD Pharmingen). Second, cell surface markers were stained with FITC - anti-CD4 (Biolegend) antibody, followed by fixation and permeability at 4°C for 30 min in the dark using Cytofix/Cytoperm Soln Kit (BD Pharmingen). Finally, APC-anti-IFN-γ (Biolegend) and PE-anti-IL-4 (BD Pharmingen) antibodies were stained in osmotic buffer at 4°C for 30 min in the dark to detect intracellular markers. The normal control group was homologous. Then, flow cytometry was conducted, and the data were analyzed using the FACS Canto Ⅱ (Becton Dickinson) and FlowJo V10 software.

### 2.10. Western blotting

Mice lungs were crushed and lysed in radioimmunoprecipitation (RIPA) lysis buffer added with 1% protease inhibitors (Beyotime, Shanghai, China) to prepare proteins. Proteins in the BECs vitro models were also prepared in the same buffer. We measured the protein concentration by bicinchoninic acid (BCA) assay according to the manufacturer's instructions (Beyotime, Shanghai, China). We transferred the 30 μg of protein to the membrane after 1-1.5h of sodium salt dodecyl sulfate-polyacrylamide gel electrophoresis (SDS-PAGE), and Tris-buffered saline-Tween 20 (TBST) is used to wash the membrane for 5mins and then finally sealed by 5% skim milk powder for 1.5h at room temperature. After that, the membrane was incubated with appropriately diluted GATA3, T-bet, β-actin (Proteintech, USA), Runx3 and GAPDH (Abcam, USA) antibodies at 4°C overnight. Then, the membrane was incubated with the secondary antibody at room temperature for 1h. We get the images with a chemiluminescence gel imaging system and Image J software (National Institutes of Health) and measured the band intensities. The relative protein expression level was calculated by the ratio of the gray value of target bands to that of GAPDH or β-actin 26, 27.

### 2.11. Immunofluorescence

BECs were adherent to chamber slides. Specimens were blocked in blocking buffer for 60 min. The blocking solution was aspirated and diluted anti-keratin antibody was applied (1:100, Abcam, USA) and incubated overnight at 4°C. The specimens were rinsed three times in 1× PBS (5 min each). The specimens were incubated in secondary antibody (1:100, Abcam, USA) and maintained for 2h at room temperature in the dark, then rinsed three times in 1× PBS (5 min each). The cover slipped slides were sealed using ProLong Gold Antifade Reagent with DAPI (Abcam, USA).

### 2.12. Enzyme-Linked Immunosorbent Assay (ELISA)

Blood serum of volunteers and mice were gathered for ELISA. The levels of DHT (Dihydrotestosterone, an Androgen), E2 (17β-Estradiol, an Estrogen) and DHT/E2 ratio were measured by ELISA kits (CSB-E07878h and CSB-E07878m for human and murine serum DHT measurements, respectively. CSB-E07286h and CSB-E07286m or human and murine serum E2 measurements, respectively) according to the manufacturer's instructions 28, 29. The supernatants of cell cultures were collected to measure IFN-γ and IL-4 using ELISA kits following the instructions provided by the manufacturer (CSB-E04578m and CSB-E04634m for IFN-γ and IL-4 measurements, respectively) 30.

### 2.13. Statistical analyses

Quantitative data were recorded as Mean±SD and were analyzed using GraphPad Prism8 (San Diego, CA, USA). Differences were assessed using the unpaired Student's t-test between two groups, and one-way analysis of variance with Tukey's multiple comparison test among three or more groups. Qualitative data were analyzed with chi-square test. P < 0.05 was considered to represent significance.

## 3. Results

### 3.1. Asthma patients have decreased serum DHT concentrations and elevated E2 concentrations

**Figure 1:**
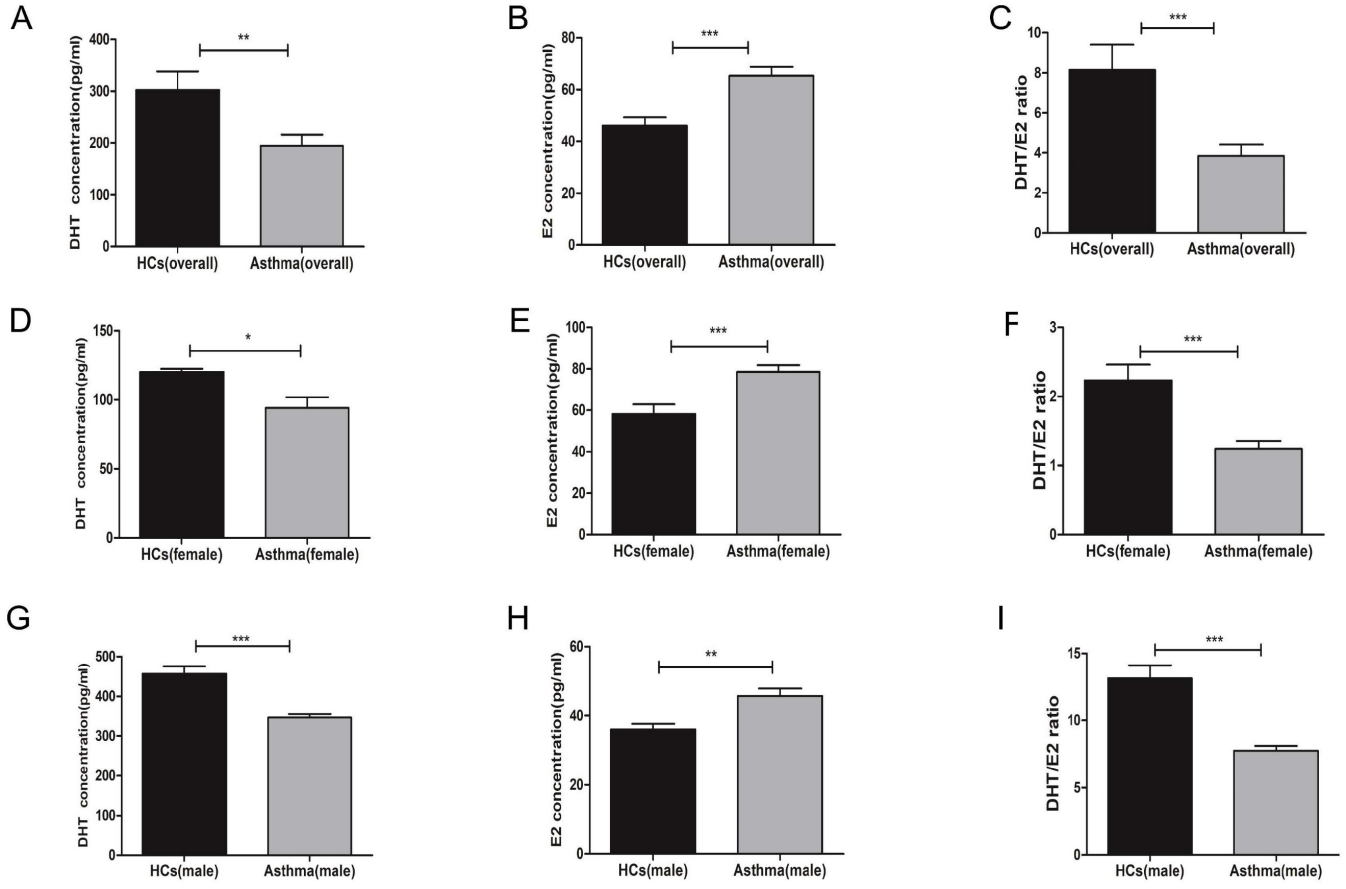
Serum DHT, E2 and DHT/E2 ratios in HCs and asthma patients. (A, B and C) The concentrations of DHT, E2 and DHT/E2 ratios were detected in HCs and asthma patients by ELISA, (D, E and F) The concentrations of DHT, E2 and DHT/E2 ratio were detected in female HCs and female asthma patients, (G, H and I) The concentrations of DHT, E2 and DHT/E2 ratio were measured in male HCs and male asthma patients. Comparisons were performed by t test. DHT, dihydrotestosterone; E2, estradiol; DHT/E2, the ratio of DHT/E2; HCs, healthy controls. * p<0.05, ** p<0.01, *** p<0.001.

First, we measured the serum concentrations of sex hormones in healthy controls and asthma patients. A total of 59 participants were enrolled, including 27 men (13 healthy controls and 14 asthma patients) and 32 women (11 healthy controls and 21 asthma patients). The demographic characteristics and lung function indexes were displayed in Table 1. Overall, there were no significant differences on age, sex, and body mass index (BMI) between the healthy control group and asthma group, while the forced expiratory volume in the first second (FEV1), forced vital capacity (FVC), and FEV1/FVC were significantly decreased in asthma patients. When compared to healthy controls, asthma patients had decreased serum concentrations of DHT and increased serum concentrations of E2 (Figure 1A-C). To avoid bias introduced by sex difference, we compared the levels of serum sex hormones between healthy controls and asthma patients in each sex category. Both male and female asthma patients had decreased DHT concentrations and elevated E2 concentrations, and the DHT/E2 ratios were significantly lowered in the asthma group (Figure 1D-I).

### 3.2. Mice with experimental asthma had decreased serum DHT concentrations and elevated E2 concentrations, and combination of DHT plus E2 alleviated airway inflammation and resistance

**Figure 2:**
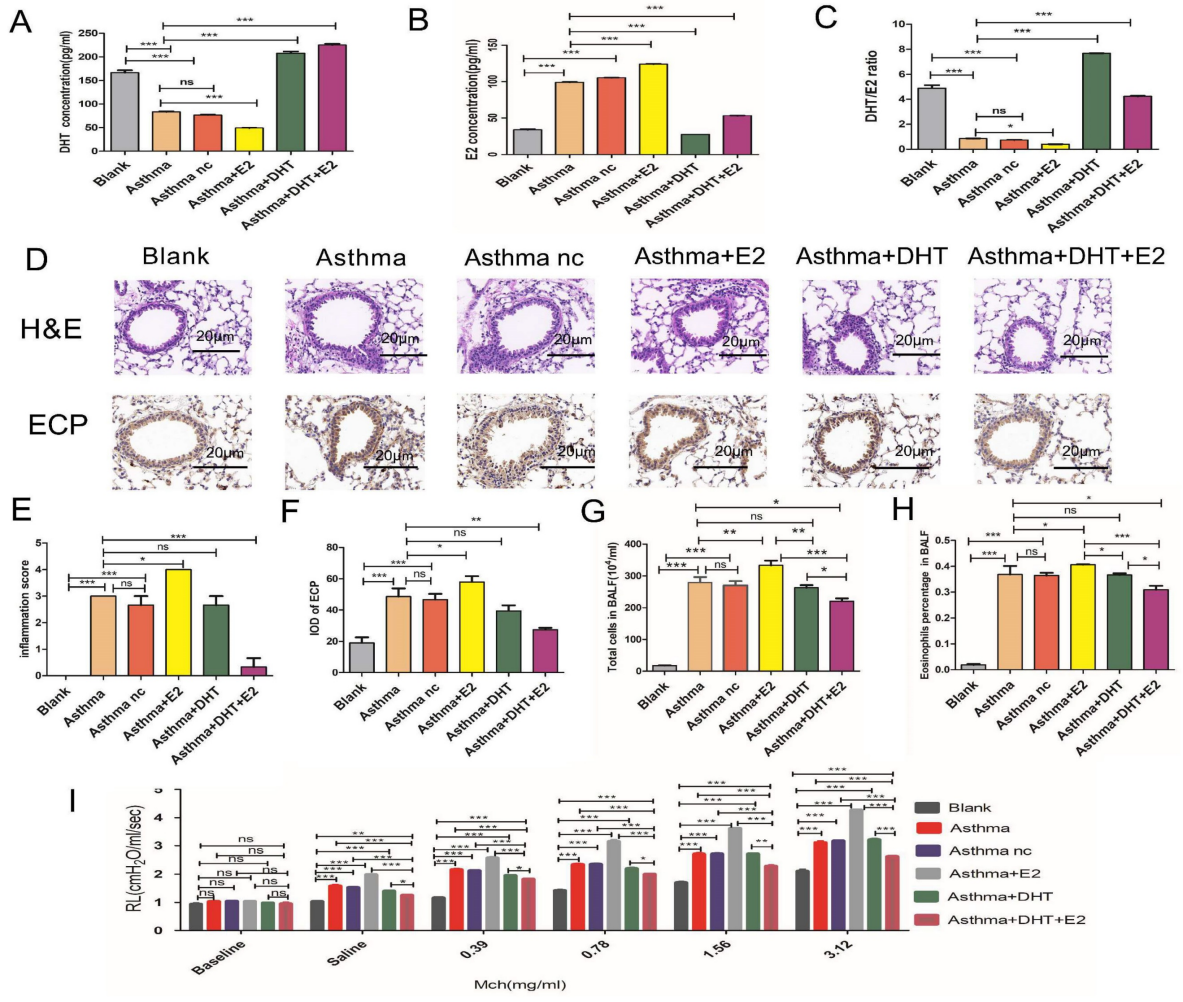
Combination of androgen and estrogen affected airway inflammation and resistance in asthma. (A, B and C) The concentrations of DHT, E2 and DHT/E2 ratio in the blank, asthma, asthma nc, asthma+E2, asthma+DHT and asthma+DHT +E2 groups. (D) Lung tissues of each group were stained with hematoxylin and eosin stain (H&E) and immunohistochemistry eosinophil antibody (anti-ECP). Scale bar = 20μm. (E and F) Inflammatory cell infiltration scores, and quantification of ECP staining integrated optical density (IOD) in lung tissues. (G and H) Total cells, and eosinophil cells in bronchoalveolar lavage fluid in all groups. (I) Lung resistance in different experimental groups. Mch, methacholine; ns, no significance. * p < 0.05. ** p < 0.01. *** p < 0.001.

We have found disturbed sex hormone balances among asthma patients both in men and women, we next tend to explore whether hormone intervention could influence asthmatic pathophysiological changes. We established murine models for asthma according to previous studies 13-15. Similar to the findings observed in human participants, we have found that the serum DHT concentrations decreased significantly in mice with experimental asthma, while the E2 concentrations elevated comparing to control mice (Figure 2A-C). Significant airway inflammation and resistance were induced in mice with experimental asthma, evidenced by infiltration of inflammatory cells (especially eosinophils) in the peri-bronchial regions and increased pulmonary resistance (RL) (Figure 2D-I). Intra-peritoneal injection of DHT or E2 significantly elevated the serum concentrations of the hormones, but administration of a single hormone (neither DHT nor E2) had no significant influences on airway inflammation and resistance. However, administration of DHT plus E2 markedly reduced peri-bronchial inflammatory cell infiltration and pulmonary resistance (Figure 2A-I). These results suggested that combination of DHT and E2 could alleviate airway inflammation and resistance in asthma.

### 3.3. Combination of DHT plus restored Th1/Th2 imbalance and Runx3 expression in mice with experimental asthma

**Figure 3:**
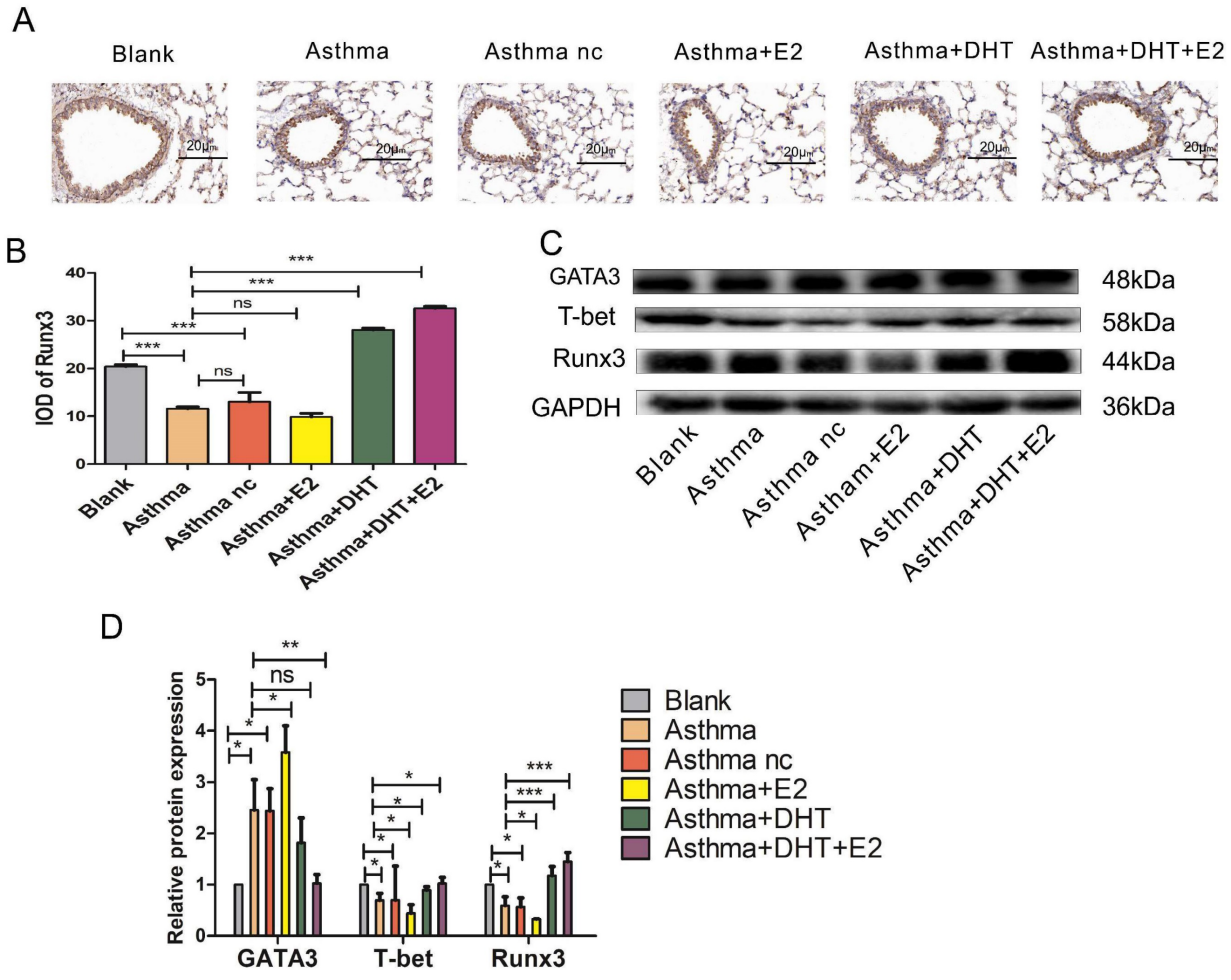
The expression of Runx3 and balance of Th1/Th2 was influenced by combination of androgen and estrogen in asthma. (A) Lung tissues of each group were stained with immunohistochemistry for anti-Runx3. Scale bar = 20μm. (B) quantification of Runx3 staining integrated optical density (IOD) in lung tissues. (C and D) GATA3, T-bet, and Runx3 protein expression in lung tissues was detected by western blotting, and GAPDH served as an internal control. ns, no significance. * p < 0.05. ** p < 0.01. *** p < 0.001.

**Figure 4:**
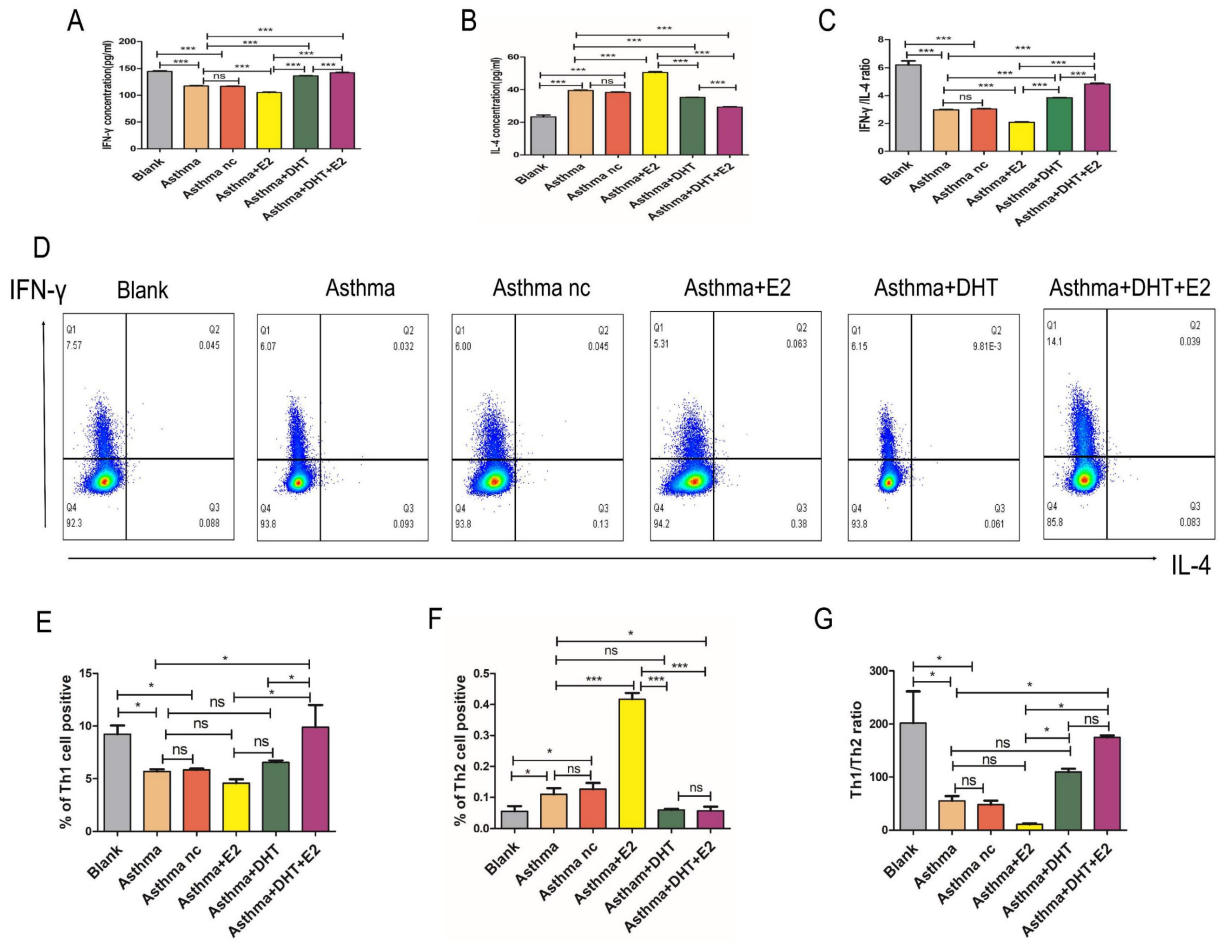
Combination of androgen and estrogen affected the expression of IFN-γ, IL-4 and Th1/Th2(IFN-γ/IL-4) cells in asthma. (A-C) The serum levels of IFN-γ, IL-4, and IFN-γ/IL-4 in each group were detected by ELISA. (D-G) Detection and measurement of Th1 and Th2 cells by flow cytometry from the different experimental mouse splenocytes. ns, no significance. * p < 0.05. ** p < 0.01. *** p < 0.001.

Next, we tend to explore the underlying mechanisms of sex hormones on asthmatic airway inflammation. It is well-characterized that disturbed Th1/Th2 balance contribute to the pathogenesis of asthma 11, and Runx3 participates in the differentiation of Th cells 19, 20, therefore we hypothesize that sex hormones might influence asthmatic airway inflammation via modulating Th1/Th2 balance and Runx3 expression. We applied both IHC and Western Blotting to detect the expression of Runx3 in the lungs of mice with experimental asthma. The expression of Runx3 decreased drastically in asthma groups, administration of E2 did not significantly alter the expression of Runx3, while DHT markedly restored its expression. Moreover, combination of DHT and E2 further enhanced the expression of Runx3 in the lungs of mice with experimental asthma (Fig. 3A-D). IFN-γ and IL-4 are representative cytokines of Th1 and Th2 cells, T-bet and GATA3 are the master transcription factors for Th1 and Th2 cell differentiation, respectively 31. The expression of T-bet significantly decreased in the lungs of asthmatic mice, while the expression of GATA3 drastically elevated, indicating a shift toward Th2 differentiation. Administration of E2 further lowered T-bet expression and increased GATA3 expression, and DHT intervention had the opposite effects. Furthermore, combination therapy with DHT plus E2 almost restored the expressions of T-bet and GATA3 to baseline levels (Figure 3C-D). Results on serum IFN-γ and IL-4 concentrations and Th1/Th2 percentages in spleenocytes showed the similar trends (Figure 4A-G). Altogether, these results suggested that sex hormones could restore the Th1/Th2 imbalance seen in asthma, with combination of DHT plus E2 had the highest efficacy.

### 3.4. Combination of DHT plus E2 modulated Runx3 expression in BECs and potentiated Th1 differentiation

**Figure 5:**
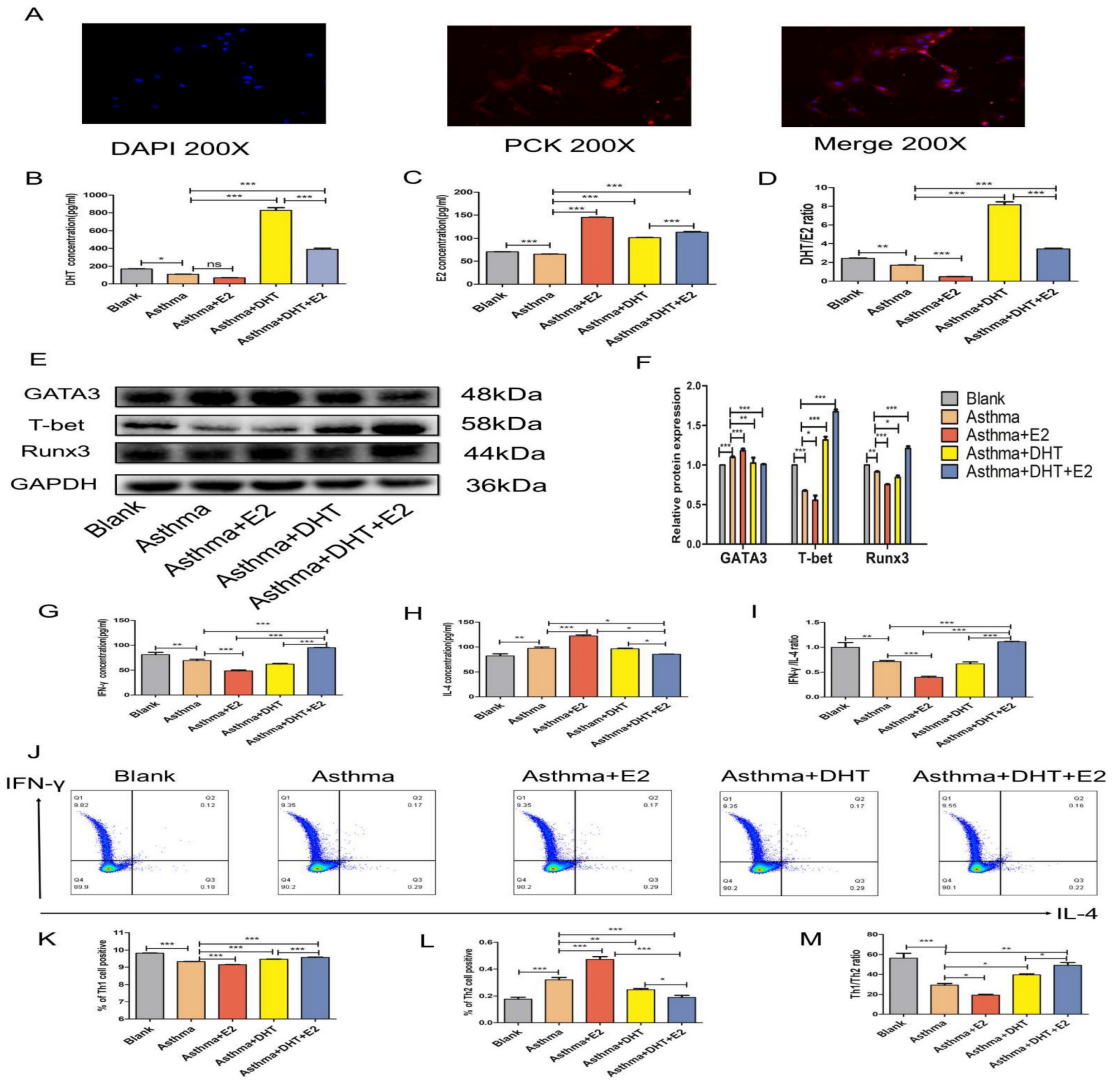
Combination of androgen and estrogen promoted the expression of Runx3 in bronchial epithelial cells (BECs), and the expression of Th1 cells from CD4+ naïve T cell co-cultured BECs. (A) The positive rate of cytokeratin in BECs was detected by immunofluorescence. (B, C, and D) The concentrations of DHT, E2 and DHT/E2 in supernatant of each group were measured by ELISA. (E and F) GATA3, T-bet, and Runx3 protein expression in BECs was detected by western blotting, and GAPDH served as an internal control. (G-I) The concentrations of IFN-γ, IL-4 and IFN-γ/IL-4 in each group were detected by ELISA. (J-M) The expression of Th1 and Th2 cells was measured by flow cytometry. ns, no significance. * p < 0.05. ** p < 0.01. *** p < 0.001.

In our previous studies, we have found that bronchial epithelial cells (BECs) could contribute to the pathogenesis of asthma via affecting the differentiation of T helper cells 13-15, 28, we next sought to determine whether sex hormones intervention influence Th1/Th2 balance and the possible underlying mechanisms. DHT, E2, or combination of DHT and E2 were applied to intervene primary murine BECs and CD4+ naïve T cell co-culture systems 32 (Figure 5A). The DHT level in supernatants increased significantly in asthma+DHT and asthma+DHT+E2 groups compared with asthma group, while there was no significant difference between asthma and asthma+E2 group (Figure 5B). The E2 level increased significantly in asthma+E2, asthma+DHT and asthma+DHT+E2 groups compared with asthma group (Figure 5C). The DHT/E2 ratio of asthma+DHT and asthma+DHT+E2 groups were significantly higher than those in asthma group (Figure 5D). The expression of Runx3 in BECs increased significantly in asthma+DHT+E2 and asthma+DHT groups compared with asthma group. In addition, T-bet and Runx3 showed the same trend, while GATA3 showed the opposite trend (Figure 5E&F). Furthermore, we measured the levels of IFN-γ and IL-4 in the supernatants by ELISA, and detected Th1 and Th2 cell counts by flow cytometry. IFN-γ concentration, IFN-γ /IL-4 ratio, Th1 cell counts, and Th1/Th2 ratio in asthma+DHT+E2 group were significantly higher than that in asthma, asthma+E2, and asthma+DHT groups. IL-4 and Th2 cell counts in asthma+DHT+E2 group were lower than that in asthma and asthma+E2 groups, while IL-4 and Th2 cells were increased in asthma +DHT group, but there was no significant difference between asthma +DHT+E2 group and asthma +DHT group (Figure 5G-M). Taken together, these data indicated that combination of DHT and E2 could up-regulate the expression of Runx3 in BECs and potentiate Th1 differentiation.

### 3.5. Combination of DHT plus E2 modulated Th differentiation via regulating Runx3 in BECs

**Figure 6:**
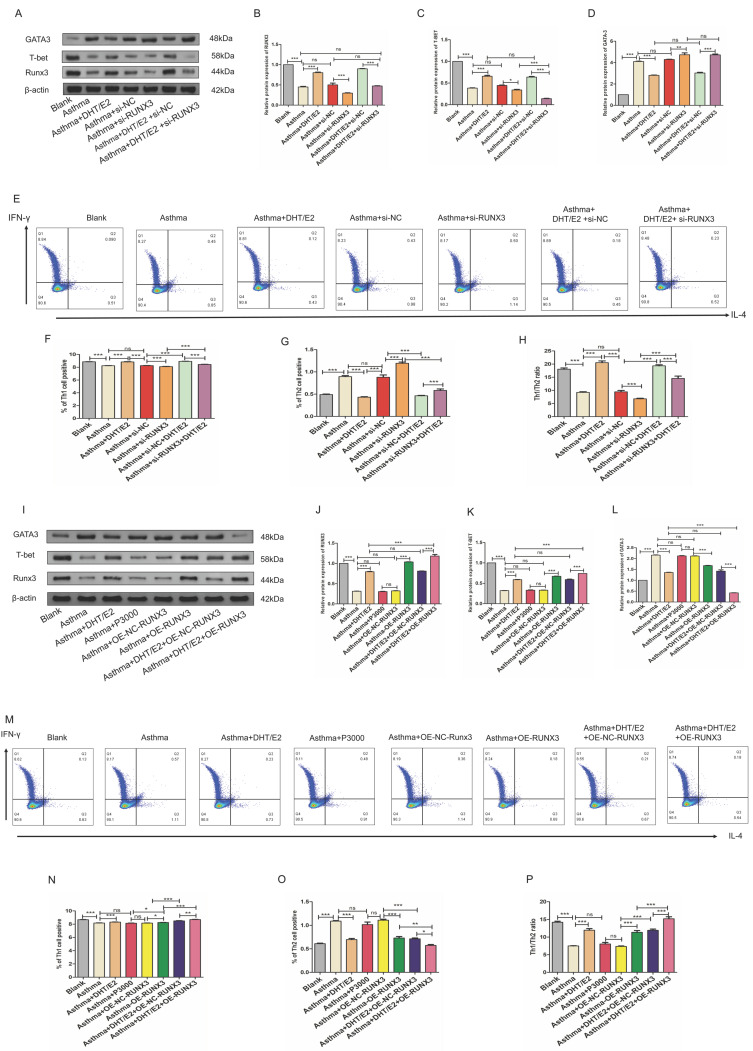
Combination of androgen and estrogen regulated Th differentiation by mediated Runx3 expression. (A-D) Western blotting was used to detect the expression of Runx3, T-bet, and GATA3 of each group with or without Runx3 knockdown, and β-actin served as an internal control. (E-H) Detection and measurement of Th1 and Th2 cells by flow cytometry. (I-L) Western blotting was used to detect the expression of Runx3, T-bet, and GATA3 of each group with or without Runx3 overexpression, and β-actin served as an internal control. (M-P) Detection and measurement of Th1 and Th2 cells by flow cytometry. ns, no significance. * p < 0.05. ** p < 0.01. *** p < 0.001.

To further explore whether sex hormones potentiate Th1 differentiation through modulating the expression of Runx3 in BECs, we knocked down or overexpressed Runx3 in BECs and then applied combination of DHT plus E2 to BEC- CD4+ naïve T cell co-culture systems. Similar to the results presented above, expression of Runx3 in BECs decreased in the asthma group, which was further lowered with Runx3 knockdown. Treatment with DHT and E2 significantly restored the expression of Runx3 in BECs, but these effects were abrogated by Runx3 siRNA (Figure 6A&B). Expression of GATA3, the master transcription factor of Th2, increased drastically in the asthma group. Treatment with DHT and E2 significantly reduced the elevation of GATA3 expression, which was abrogated by Runx3 knockdown (Figure 6A&C). Expression of T-bet, the master transcription factor of Th1, decreased substantially in the asthma group. Treatment with DHT and E2 significantly restored T-bet expression, which was also mitigated by Runx3 knockdown (Figure 6A&D). These results were further supported by flow cytometry analyses (Figure 6E-H). Taken together, these data suggested that knockdown of Runx3 significantly abated the effects of DHT and E2 combination on Th cell differentiation. On the other hand, overexpression of Runx3 in BECs further enhanced the expression of T-bet restored by DHT and E2 combination, while mitigated the expression of GATA3 (Figure 6I-L). Flow cytometry analyses also confirmed that overexpression of Runx3 in BECs further promoted Th1 differentiation potentiated by DHT and E2 combination (Figure 6M-P). Overall, these results indicated that combination of DHT plus E2 modulated Th cell differentiation via modulating the expression of Runx3 in BECs.

## 4. Discussion

Asthma is a heterogeneous chronic airway inflammatory disease with a rising global incidence rate, and there is a significant discrepancy in epidemiology between male and female patients. Women are more likely to develop asthma and prone to have more severe phenotypes than man 2, and sex hormones are considered to be associated with this gender difference 3. The hormonal levels fluctuate in women during menstrual cycles, pregnancies, the perimenopausal and menopause periods, which are correlated to higher risk of asthma exacerbations and worsened symptoms 4, 5, 33, and it's been well-characterized that reproductive factors participated in the development of asthma 34. Different sex hormones have varied influences on asthma. Estrogen includes estrone, estradiol, estriol, and estretrol, with estradiol (E2) being the most potent and prevalent hormone. Estrogens have dual effects on the development of asthma, it could either ameliorate or aggravate airway inflammation 35, 36, and estrogen supplementation with hormone replacement therapy has been reported to be associated with elevated or reduced risk of asthma development 37, 38. Estrogen is previously believed to exert its effect as a nuclear receptor, but recent studies have revealed the existence of G-protein coupled estrogen receptor (GPER) on the plasma membrane, which might be a possible explanation for the conflicting effects of estrogen on asthma 39. Androgens, on the other hand, are reported to exert anti-asthma effects. Low levels of endogenous anabolic androgens are associated with poorer asthma control in female patients 40, and androgens could alleviate both Th2 and Th17 mediated airway inflammations seen in asthma 41, 42, implicating its potential therapeutic application. In the present study we have found that combination of estrogen (E2) and androgen (DHT) have better effects on mitigating airway inflammations in asthma than single use of either hormone. Similar synergistic effects of estrogen and androgen have been reported before 43-45, yet the underlying mechanisms remain unclear, further explorations are called for to clarify this phenomenon.

Our previous studies have suggested a contributing role of BEC-T cell interplay in the pathogenesis of asthma 13-15, and recent studies have reported that bronchial epithelial cells could express androgen receptor 46 and estrogen receptor 46. Moreover, it's been reported that expression levels of androgen receptor in BECs correlates with asthma severity 47, 48, and activation of GPER on BECs could maintain epithelial integrity and alleviate allergic asthma 39. In the present study, we have found that combination of DHT and E2 shifted the differentiation of T cell toward Th1 via restoring the expression of Runx3 in BECs. Our findings could help us to better understand how sex hormone receptor signaling in BECs participate in the pathogenesis of asthma.

Runx3 is a member of the Runx family proteins and had been found to be essential in T cell functions. Runx3 could regulate the Th1/Th2 balance and airway inflammatory responses in asthma 19, 20. It's been reported that estrogen treatment could lead to Runx3 gene hypermethylation and decreased expression in epithelial cells 49, in the present study we have also found that treatment with E2 alone reduced the expression of Runx3 in BECs while treatment with DHT alone increased its expression, and combination of DHT plus E2 induced higher expression of Runx3 than DHT alone. These results suggest that combination of DHT and E2 not only “override” the effect of E2 alone, but also enhanced the effect of DHT. The mechanism for this phenomenon is beyond the scope of the current study, our hypothesis is that the existence of two types of estrogen receptor (plasma membrane bound GPER and nuclear receptor) might be responsible for these paradoxical effects of E2: in the presence or absence of DHT, E2 might exert its functions via different receptor signaling pathways, one pathway could repress the expression of Runx3 while the other pathway could promote the expression of Runx3. Further studies are needed to clarify whether our hypothesis is valid or not.

There are several other limitations to the present study. First, we used a single murine model of asthma, the findings were not validated on human samples. Second, the dosage of either DHT or E2 used in animal experiments and in vitro assays could not be directly translated into clinical applications. Finally, the exact mechanisms of how Runx3 expression in BECs influence the interplay between BECs and T cells remain unstudied. These unanswered questions should be the topic of our further investigations.

## Figures and Tables

**Table 1:**
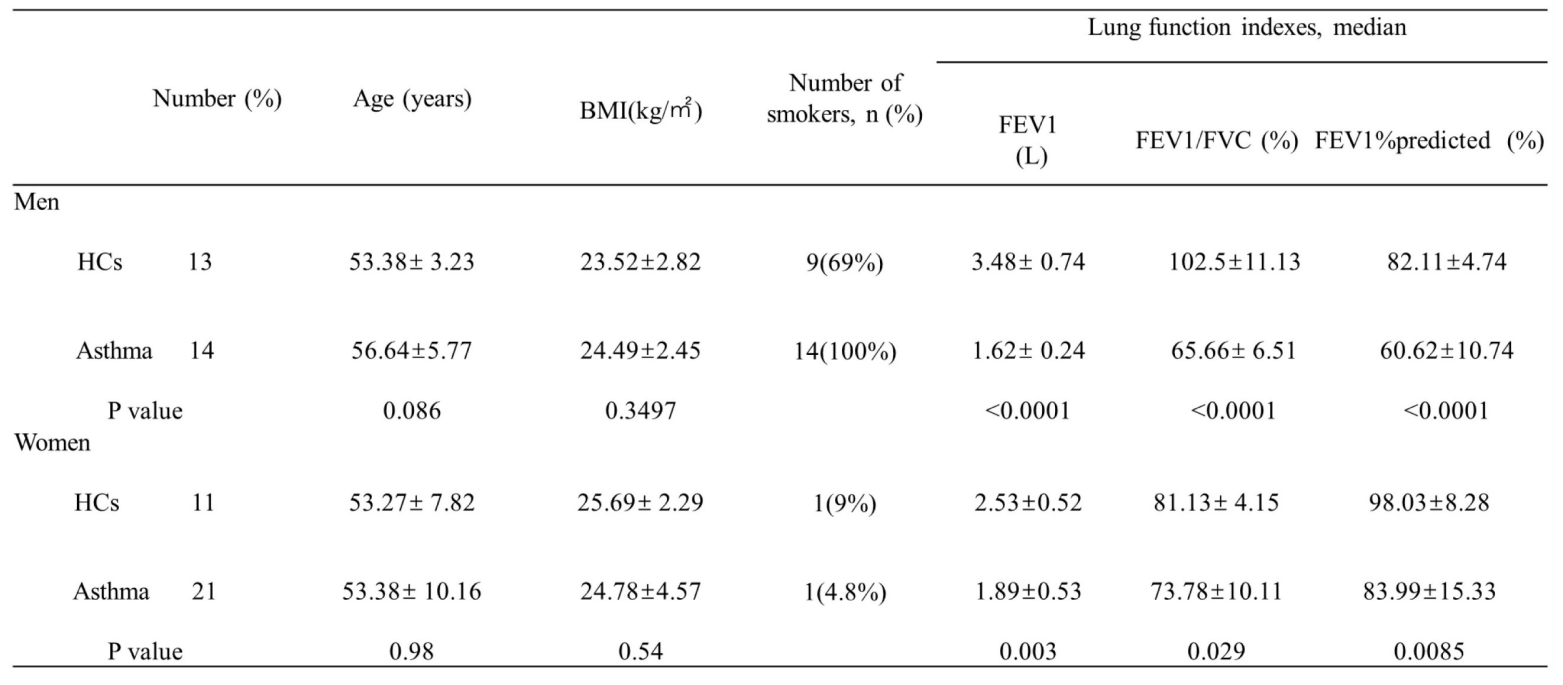
Subject characteristics

BMI, body mass index; FEV1, forced expiratory volume in the first second; FVC, forced vital capacity; HCs, healthy controls.
